# FSP1/S100A4-Expressing Stem/Progenitor Cells Are Essential for Temporomandibular Joint Growth and Homeostasis

**DOI:** 10.1177/00220345251313795

**Published:** 2025-02-14

**Authors:** T. Tuwatnawanit, W. Wessman, D. Belisova, Z. Sumbalova Koledova, A.S. Tucker, N. Anthwal

**Affiliations:** 1 Centre for Craniofacial and Regenerative Biology, Faculty of Dentistry, Oral and Craniofacial Sciences, King’s College London, London, UK; 2 Department of Conservative Dentistry and Prosthodontics, Faculty of Dentistry, Srinakharinwirot University, Wattana, Bangkok, Thailand; 3 Faculty of Medicine, University of Helsinki, Helsinki, Finland; 4 Faculty of Medicine, Masaryk University, Brno, Czech Republic; 5 Laboratory of Tissue Morphogenesis and Cancer, Institute of Molecular Genetics of the Czech Academy of Sciences, Prague, Czech Republic

**Keywords:** condyle, fibrocartilage, osteoarthritis, stem cell, Wnt signaling, temporomandibular disorders

## Abstract

The temporomandibular joint (TMJ) is one of the most used joints in the body. Defects and wear in the cartilage of the joint, condyle, and fibrocartilage disc lie at the heart of many common TMJ disorders. During postnatal development, the condyle acts as a growth center for the mandible, with cells moving as a conveyor belt away from the top of the condyle as they differentiate. The superficial layers of the condyle have been proposed to contain stem/progenitor populations to allow growth and maintain homeostasis. Here we have focused on the role of fibroblast-specific protein 1 (FSP1; also known as S100a4) as a key fibroblast stem/progenitor marker for the condyle. Lineage tracing with *FSP1-Cre;R26RmTmG* mice revealed that FSP1-expressing cells were restricted to the superficial fibroblast zone, giving rise to all layers of the condyle over time. The FSP1-expressing cells overlapped with other putative stem cell markers of the condyle, such as Gli1 and scleraxis. BrdU pulse chase experiments highlighted that a subset of FSP1 fibrocartilage was label retaining, suggesting that FSP1 labels a novel stem/progenitor cell population in the condyle. Destruction of FSP1-expressing cells by conditional diphtheria toxin activity in *FSP1-Cre;R26RDTA* mice resulted in severe TMJ osteoarthritis with loss of the cartilage structure. Lgr5-expressing cells in the superficial layer of the condyle have been shown to create a Wnt inhibitory niche. FSP1 expression postnatally was associated with a reduction in canonical Wnt activity in the condyle. Importantly, constitutive activation of Wnt/β catenin in FSP1-expressing cells led to a downregulation of FSP1 and progressive postnatal loss of TMJ condylar hyaline cartilage due to loss of the superficial stem/progenitor cells. These data demonstrate a novel role for FSP1-expressing cells in the superficial zone in growth and maintenance of the TMJ condylar cartilage and highlight the importance of regulating Wnt activity in this population.

## Introduction

The temporomandibular joint (TMJ) is one of the most frequently utilized joints in the human body, comprising the condylar process of the mandible (lower jawbone) and the glenoid fossa of the temporal bone (upper jaw). A fibrous disc within a synovial capsule lies between these structures, serving as a cushioning element. The TMJ articular surface is covered with fibrocartilage, a dense and avascular connective tissue rich in collagen types I and II, which resembles a mix of fibrous and hyaline cartilage (Benjamin and Ralphs 2004; Delatte et al. 2004; Lowe and Almarza 2017). This fibrocartilage provides a functional buffer between the bony surfaces of the condyle and fossa, accommodating the extensive range of TMJ movements (Wadhwa and Kapila 2008).

TMJ osteoarthritis (TMJOA) poses a significant clinical challenge due to the erosion of TMJ cartilage. The limited regenerative capacity of fibrocartilage leads to condyle and fossa articular cartilage degradation, inflammatory subchondral bone remodeling, synovitis, and extracellular matrix degradation, as well as clinical symptoms including pain, joint noises, and impaired jaw function (Wang et al. 2015; Cardoneanu et al. 2022). TMJOA, the most severe form of temporomandibular disorder, predominantly affects women and accounts for over half of temporomandibular disorder cases (Alzahrani et al. 2020).

The condylar process develops as a secondary cartilage during embryogenesis, integrating with the dentary bone and serving as a growth center in postnatal life (Frommer 1964). The mature mandibular condyle is structured into 4 zones: a superficial fibrous tissue, a prechondroblastic zone (expressing SOX9), a chondroblastic zone (expressing collagen II), and a hypertrophic zone (expressing collagen X; Shibukawa et al. 2007), with cells moving as a conveyor belt through the zones as they differentiate. The superficial fibroblast tissue, which forms the articular surface and interfaces with the disc, has been suggested to house a stem cell population with canonical Wnt signaling crucial for maintaining fibrocartilage homeostasis (Embree et al. 2016; Ruscitto et al. 2023). Lineage tracing has highlighted several putative stem cell markers, including *Gli1* and *(Scx)* (Ma et al. 2021; Lei et al. 2022; Bi et al. 2023).

Fibrocartilage, lining the TMJ articular surfaces, has unique molecular features as compared with hyaline or elastic cartilage, notably high levels of collagen I, and the expression of fibroblast-specific protein 1 (FSP1), also known as S100A4 (Delatte et al. 2004; Park et al. 2015). FSP1, a member of the S100 family of calcium-binding proteins, plays a crucial role in various diseases: fibrosis, cirrhosis, pulmonary diseases, cardiac hypertrophy, neuronal injuries, and cancer (Strutz et al. 1995; Schneider et al. 2008). These conditions commonly involve fibrosis, tissue remodeling, epithelial-mesenchymal transition, and inflammatory mechanisms (Schneider et al. 2008). FSP1 is expressed in a variety of cell types, such as fibroblasts, endothelial cells, smooth muscle cells, immune cells, bone, and chondrocytes associated with elastic cartilage and fibrocartilage (Strutz et al. 1995; Tsutsumi et al. 2009; Teng et al. 2011; Szabo et al. 2019; Chen et al. 2021). FSP1 performs diverse functions in the nucleus, cytoplasm, and extracellular space, acting intracellularly and as an extracellular paracrine molecule affecting cell motility, viability, differentiation, and contractility (Schneider et al. 2008; Boye and Mælandsmo 2010; Teng et al. 2011). *Fsp1* knockout mice had mild bone phenotypes and exhibited altered bone density due to decreased bone resorption activity (Erlandsson et al. 2013; Kim et al. 2021). Recent research has utilized FSP1 as a fibrochondrocyte marker to differentiate fibrocartilage, with a focus on the TMJ disc (Park et al. 2015; Su et al. 2020). However, the specific role of FSP1 in the structure of the TMJ remains unexplored.

In this study, we identify FSP1-expressing fibroblasts in the superficial layer of the TMJ as stem/progenitor cells crucial for condylar cartilage growth and joint homeostasis. This finding demonstrates that FSP1-expressing cells are essential for TMJ development and maintenance, offering a novel model for studying osteoarthritis in the TMJ.

## Materials and Methods

### Animal Preparation

*FSP1-Cre* mice (Bhowmick 2004) were mated to 3 strains of mice—*R26RmTmG* (referred to as *mTmG*; Muzumdar et al. 2007), *R26RDTA* (referred to as *DTA*; Ivanova et al. 2005), and *Ctnnb1*^ex3(loxP)^ (referred to as *βcatGOF*; Harada 1999)—to generate transgenic mice for lineage tracing through Cre driven green fluorescent protein, lineage depletion through Cre driven diphtheria toxin (DTA) expression, and gain-of-function studies through Cre driven *β catenin stabilization* under the approval of the Ministry of Education, Youth and Sports of the Czech Republic (license MSMT-24093/2021-3) and the supervision of the Expert Committee for Laboratory Animal Welfare of the Faculty of Medicine, Masaryk University, at the Laboratory Animal Breeding and Experimental Facility (license 310 58013/2017-MZE-17214). Transgenic animals were maintained on a C57BL/6 background. This work was carried out under UK Home Office license and regulations in line with the regulations set out under the United Kingdom Animals (Scientific Procedures) Act 1986 and complied to ARRIVE 2.0 guidelines. The mice (*n* = 80) were housed in individually ventilated or open cages, all with an ambient temperature of 22 °C, a 12h:12h light:dark cycle, and food and water ad libitum. Details of all mice used are listed in Appendix Table 1.

*Axin2-CreERT2;tdTom* mice were intraperitoneally injected with tamoxifen (0.15 mg/g of body weight) at postnatal day 16 (P16) and culled at P18. For 5-bromo-2′-deoxyuridine (BrdU) labeling, pregnant CD1 mice were intraperitoneally injected with 20 mg/kg of BrdU at embryonic day 17.5 (E17.5) and E18.5, and the pups were culled at P2, P21, and P48. *Mesp1Cre;tdTom* mice were collected at P21.

### TMJ Culture

P21 CD1 condylar heads were dissected, and contralateral sides were cultured in Advanced DMEM/F12 with the addition of either 50µM BIO in DMSO, or a equivalent volume of DMSO as a control following a modified Trowell method (Gaete and Tucker 2013).

### Tissue Preparation for Micro–computed tomography Scanning and Immunohistochemistry Staining

Tissue samples were fixed in 4% paraformaldehyde and washed with phosphate-buffered saline (PBS). Skull samples were placed into a 19-mm tube containing 70% ethanol and scanned by a SCANCO MEDICAL µCT 50 scanner (70 kV, 114 µA, 20 µm). The TMJ was reconstructed in 3 dimensions via Amira and MeshLab software.

Samples were decalcified in 0.5M EDTA, dehydrated, embedded in paraffin, and sectioned at 6 µm in the frontal plane with a Leica RM2245 microtome (Leica Biosystems). Sections from the central TMJ, as identified by surrounding tissue landmarks, were then mounted in parallel sequence on TruBOND380 slides (Matsunami).

### Histology Staining and TUNEL Assay

Serial tissue sections of the TMJ were stained with trichrome (picrosirius red, alcian blue, and hematoxylin), hematoxylin and eosin, and safranin O staining for histologic investigation (Appendix Fig. 1). A TUNEL assay (TdT-mediated dUTP nick end labeling) was performed with the In Situ Apoptosis Detection Kit (TaKaRa) following the manufacturer’s instructions. A NanoZoomer 2.0-HT Digital Slide Scanner (Hamamatsu Photonics) with NDP.view2 software was used to examine and capture the morphology of histology-stained TMJ slides.

Osteoarthritis levels in the TMJ were scored in safranin O– or trichrome-stained sections per the Osteoarthritis Research Society International scale (Laverty et al. 2010).

### Immunofluorescence and RNAscope

Immunofluorescence staining was conducted for FSP1, SOX9, type II collagen, and BrdU. Endogenous fluorescence is lost after wax processing of reporter mice, so immunofluorescence was employed via green and red fluorescent protein (Appendix Table 2). After deparaffinization with NeoClear and rehydration in a declining series of ethanol dilutions, paraffin sections were covered in trypsin at room temperature (RT) for 10 min for antigen retrieval. For collagen staining, tissue sections were enzymatically treated with chondroitinase ABC (0.1 U/mL) and hyaluronidase (0.6 U/mL) for 45 min at 37 °C. The sections were incubated for 30 min at RT by a generic blocking buffer (1% bovine serum albumin [BSA], 0.1% Triton X-100 in PBS). Sections were then treated overnight at 4 °C with primary antibodies diluted in blocking buffer in a moisture chamber. The slides were washed and incubated with secondary antibodies diluted in blocking buffer for 2 h at RT in dark. Nuclear counterstaining was performed with DAPI (Fluoroshield; Sigma-Aldrich).

RNAscope in situ hybridization (Advanced Cell Diagnostics) was used following the manufacturer’s instructions. *Axin2, Lgr5, Gli1*, *Scx*, and *Fsp1* probes were utilized (Appendix Table 2). Negative control staining was carried out (Appendix Fig. 2). Slides were imaged on a confocal microscope (ZEISS LSM 980) and ZEISS Apotome.2. Experimental data were analyzed and quantified with ImageJ and Qupath 0.5.1.

### Collagen-Hybridizing Peptide Staining

Tissue sections were deparaffinized and rehydrated. 5% Goat serum in PBS was added and incubated for 30 min at RT to block nonspecific binding. The biotin-conjugated collagen-hybridizing peptide (B-CHP) solution (15 µM, 50 µL per section) in 1% BSA was heated in the oven at 80 °C for 5 min and immediately incubated on ice for 15 s to quench the solution to RT. B-CHP solution was quickly pipetted onto each slide within 1 min and incubated overnight at 4 °C (Hwang et al. 2017). The slides were treated with Alexa Fluor 647–streptavidin solution (0.005 mg/mL) in 1% BSA for 1 h at RT. Nuclear counterstaining was performed with DAPI.

### Statistical Analysis

All experiments were replicated at least 3 times. Statistical analyses were performed with GraphPad Prism 10 software. Statistical comparisons were made among the 4 groups by 1-way analysis of variance, followed by a Tukey’s post hoc test, and 2-way analysis of variance, followed by a Sidak’s or Tukey’s multiple-comparison test. To compare the control and mutant groups, an unpaired *t* test was used. Data were expressed as the mean ± SD. A statistically significant difference was defined as *P* < 0.05.

## Results

### FSP1 Was Progressively Expressed in the Superficial Zone of the TMJ Condyle

**Figure 1.**
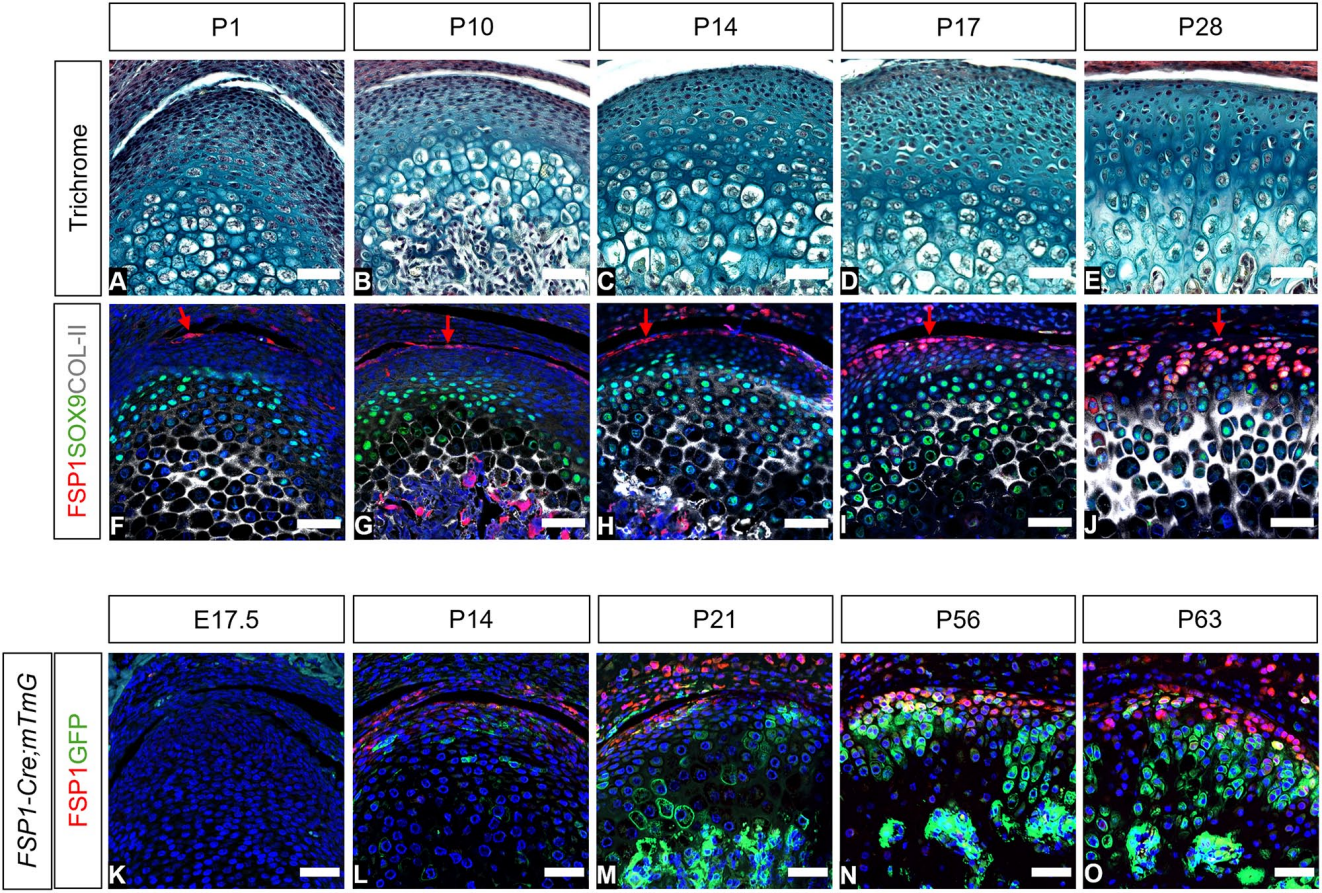
Expression and contribution of FSP1-expressing cells in the mouse mandibular condyle across development and growth. (**A**–**E**) Picrosirius red–alcian blue trichrome and (**F**–**J**) immunofluorescence staining for FSP1 (red), SOX9 (green), COL-II (gray), and DAPI (blue), illustrating the expression pattern of FSP1 during postnatal growth in CD1 mice (*n* = 3). (**F**–**J**) Arrows indicate FSP1-expressing cells (red) in the top layers of the condyle. (**K**–**O**) Lineage-tracing experiment in *FSP1-Cre;mTmG* mice (*n* = 3). Immunofluorescence staining for FSP1 was used to illustrate current FSP1 expression (red) with lineage tracing of these cells (GFP, green) and DAPI (blue). Scale bar: 50 µm. E, embryonic stage; FSP1, fibroblast-specific protein 1; GFP, green fluorescent protein; P, postnatal stage.

The expression of FSP1 was followed during murine postnatal development from birth to postweaning (Fig. 1A–E). At P1, only a few FSP1-expressing cells were associated with the top layers of the condyle at a distance from the SOX9- and collagen II–positive layers (Fig. 1F), with this number increasing as the condyle matured (Fig. 1G–I). Following weaning at P28, robust expression of FSP1, at the gene and protein levels, reached the SOX9-expressing chondrogenic zone in the condyle (Fig. 1J, Appendix Fig. 3). At E17.5, there was no FSP1 expression in the condyle, highlighting FSP1 as a postnatal marker of condylar fibroblasts (Fig. 1K).

Expression of FSP1 in the superficial zone of the condyle suggested an association with the proposed condyle stem cell niche (Embree et al. 2016). By using mesoderm tracing *Mesp1cre;TdTom* mice, expression of FSP1 was confirmed in the neural crest–derived condyle fibroblasts, rather than the mesoderm-derived invading vasculature (Appendix Fig. 4).

To investigate the contribution of the FSP1-positive cells to condyle growth, we employed *FSP1-Cre;mTmG* mice to lineage trace the FSP1 population. Immunostaining for FSP1 was used to compare current FSP1 expression (red) with lineage tracing of these cells (green; Fig. 1K–O). Postnatally, FSP1-expressing cells were observed in the top layer of the condyle, while FSP1-lineage cells expanded into the main body of the condyle (Fig. 1L, M). By P56 and P63, the *FSP1Cre*-driven reporter expression had greatly expanded, with long clones of cells reaching the hypertrophic zones and into the ossified region (Fig. 1N, O). This expansion suggested a substantial contribution of the FSP1 lineage to the mature growth of the condyle.

### *Lgr5* and *Axin2* Expression Decreased as FSP1 Switched On

**Figure 2.**
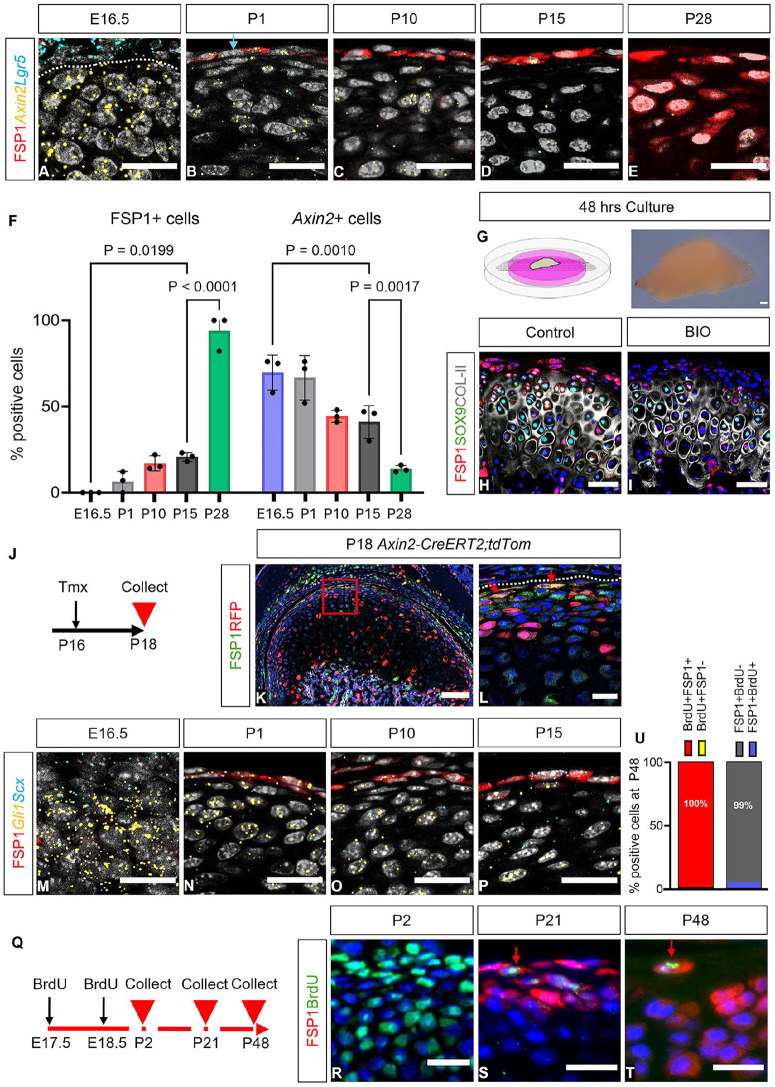
The FSP1^+^ population coexpresses putative stem cell markers. (**A**–**E**) Dual immunofluorescence and RNAscope staining for FSP1 protein (red), *Axin2* mRNA (yellow), *Lgr5* mRNA (cyan), and DAPI (gray) between E16.5 and P28 in CD1 mice (*n* = 3). (A) Dash line indicates condyle surface. (B) Arrows indicate *Lgr5*-positive, FSP1-negative cells. (**F**) Quantification of FSP1-expressing cells and *Axin2*-positive cells during condyle growth. Error bar indicates SD. *n* = 3. Two-way analysis of variance followed by Tukey’s multiple-comparison test. (**G**) At 48 h, explant culture of P21 CD1 condylar heads (*n* = 3) in ±50µM BIO in Advanced DMEM/F12. (**H**, **I**) Immunofluorescence staining for FSP1 (red), SOX9 (green), COL-II (gray), and DAPI (blue). (**J**) Tamoxifen was administered to *Axin2-CreERT2;tdTom* mice at P16 and collected at P18 (*n* = 3). (**K**) Immunofluorescence staining for FSP1 (green), RFP (red), and DAPI (blue). (**L**) The box shows a zoomed-in image; arrows indicate FSP1-expressing cells (green) coexpressing with *Axin2* (red). (**M**–**P**) Dual immunofluorescence and RNAscope for FSP1 (red), *Gli1* mRNA (yellow), *Scx* mRNA (cyan), and DAPI (gray) in different stages of CD1 mice (*n* = 3 for each stage). (**Q**) BrdU was administered to CD1 mice at E17.5 and E18.5. BrdU-positive label-retaining cells were chased in P2 (n = 3), P21 (n = 3), and P48 (n = 3). (**R**–**T**) Immunofluorescence staining for BrdU (green), FSP1 (red), and DAPI (blue). (**S**, **T**) Arrows indicate FSP1-expressing cells (red) costained with BrdU label-retaining cells (green). (**U**) The percentage of colabeled BrdU^+^ and FSP1^+^ cells at P48. Scale bars: A–E and L–T, 20 µm; H–I, 50 µm; K, 100 µm. BrdU, 5-bromo-2′-deoxyuridine; E, embryonic stage; FSP1, fibroblast-specific protein 1; P, postnatal stage; RFP, red fluorescent protein; Scx, scleraxis; Tmx, tamoxifen.

Canonical Wnt signaling is crucial for maintaining fibrocartilage homeostasis (Embree et al. 2016; Ruscitto et al. 2023). In RNAscope and immunofluorescence were employed to investigate the relationship among *Axin2*, *Lgr5*, and FSP1 from E16.5 to P28. During embryogenesis, *Lgr5* was strongly expressed in the TMJ disc and partially in the condyle, with its expression decreasing postnatally (Fig. 2A–E). Similarly, *Axin2*-positive cells were abundant throughout the condyle from embryonic stages, showing a rapid decline in the superficial layers, particularly after weaning as FSP1 levels increased (Fig. 2A–F, Appendix Fig. 5). The causal relationship between Wnt signaling and FSP1 was confirmed by condyle explant culture (Fig. 2G). FSP1 expression was lost after 48 h of culture with the Wnt/β catenin agonist BIO (Fig. 2H, I; Appendix Fig. 6). In vivo postnatally, some cells expressed *Axin2* and FSP1, suggesting that FSP1-expressing cells were in the *Axin2* lineage (Fig. 2B–D). This lineage relationship was confirmed in *Axin2-CreERT2;tdTom* mice, where red fluorescent protein/FSP1 double-positive cells were evident after tamoxifen injection (Fig. 2J–L).

### A Subset of FSP1-Expressing Cells Overlapped with Putative Stem Cell Markers and Was Label Retaining

A number of fibroblast stem cell markers have been proposed in the condyle and other fibroblast populations (Ma et al. 2021; Lei et al. 2022), with hedgehog-responsive (*Gli1* positive) and scleraxis-positive cells contributing to condylar growth (Ochiai et al. 2010; Ma et al. 2021). Interestingly, a subpopulation of FSP1-expressing cells also expressed *Gli1* and *Scx*, suggesting that these cells had stem cell–like properties (Fig. 2M–P). Retention of a BrdU label is widely used as a hallmark of stem cells (Sottocornola and Lo Celso 2012). To identify whether the FSP1-expressing cells were label retaining, pregnant mice were injected with BrdU during TMJ morphogenesis at E17.5 and E18.5 (Fig. 2Q). Confirming uptake of the label, BrdU-positive cells were observed in most condylar cells at P2 (Fig. 2R). The cells of the condyle proliferated rapidly postnatally, resulting in scant BrdU-positive cells by P21 and P48 (Fig. 2S, T; Appendix Fig. 7). By P48, very few FSP1-expressing cells were BrdU positive (Fig. 2U); importantly, all BrdU-positive cells in the top layers of the condyle were FSP1 expressing (Fig. 2S–U), suggesting that FSP1 labels a novel stem/progenitor cell population in the TMJ.

### Removal of the Superficial Layers by *Fsp1Cre*-Driven Diphtheria Toxin Led to Loss of the Condyle Structure

**Figure 3.**
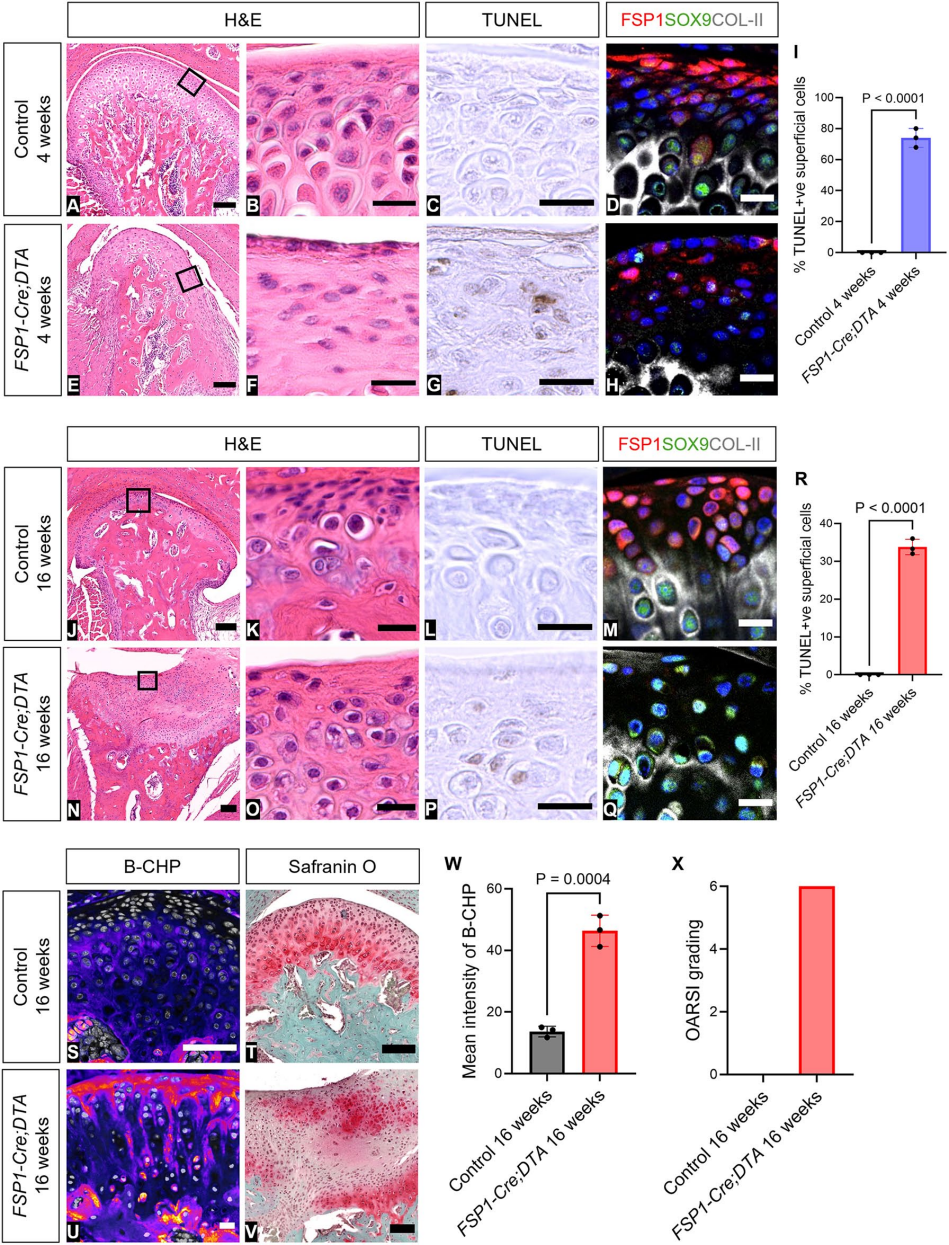
Removal of the superficial layers by *FSP1-Cre*–driven DTA led to loss of condylar structure. (**A**–**R**) H&E staining, TUNEL assay, and immunofluorescence staining in 4-wk-old (*n* = 3) and 16-wk-old (*n* = 6) *FSP1-Cre;DTA* mice with the Cre-negative *DTA* littermate controls. (A, E, J, N) H&E staining shows condyle morphology. (B, F, K, O) The box shows a zoomed-in image of superficial layer of the condyle. (C, G, L, P) TUNEL-positive cells in the superficial layers are indicated by a brown color. (D, H, M, Q) Immunofluorescence staining for FSP1 (red), SOX9 (green), COL-II (gray), and DAPI (blue). (I, R) Percentage of TUNEL-positive cells in the superficial layers of 4- and 16-wk-old *FSP1-Cre;DTA* mice as compared with the Cre-negative *DTA* littermate controls. Error bar indicates SD. *n* = 3. Unpaired *t* test. *FSP1-Cre;DTA* mice and Cre-negative *DTA* littermate controls aged 16 wk were stained with (**S**, **U**) immunofluorescence staining for B-CHP (fire LUT) and DAPI (gray) and (**T**, **V**) safranin O staining. (**W**) Mean intensity of B-CHP was analyzed with ImageJ software. Error bar indicates SD. *n* = 3. Unpaired *t* test. (**X**) OARSI grading was blindly scored by observers (*n* = 3). Scale bars: B–D, F–H, K–M, O–Q, S–U, 20 µm; A, E, J, N, T, V, 100 µm. B-CHP, biotin-conjugated collagen-hybridizing peptide; DTA, diphtheria toxin; H&E, hematoxylin and eosin; OARSI, Osteoarthritis Research Society International; TUNEL, TdT-mediated dUTP nick end labeling.

To follow the role of the superficial layers of growth and homeostasis of the condyle, these layers were selectively ablated via the *FSP1-Cre;DTA* mouse model. In the *FSP1-Cre;DTA* mouse, FSP1-expressing cells were selectively exposed to diphtheria toxin and die. The toxin cannot spread to neighboring cells, as mice do not contain the receptor for this toxin (Chang and Neville 1978). In Cre-negative *DTA* littermate control mice aged 4 and 16 wk, the condyle showed a mature pattern, with FSP1-expressing fibroblasts reaching the SOX9- and collagen-II-positive zones (Fig. 3A–D, J–M). At 4 wk, conditional ablation of FSP1-expressing cells led to a loss of the fibrocartilage layer of the condyle (Fig. 3E, F), which associated with elevated levels of apoptosis, as observed by TUNEL (Fig. 3G, I; Appendix Fig. 9) and apoptotic bodies (Appendix Fig. 8). A reduction in the number of FSP1- and SOX9-expressing cells was also noted by immunofluorescence (Fig. 3D, H). At 16 wk, a clear reduction in FSP1 expression was observed in mutants as compared with littermates, confirming the loss of a large proportion of the FSP1 population (Fig. 3P–R, Appendix Fig. 9). Hematoxylin and eosin staining highlighted a pronounced condylar structural transformation (Fig. 3N, O). Loss of the superficial layers resulted in expression of SOX9 along the surface of the condyle and diminished and disorganized distribution of collagen II (Fig. 3M, Q; Appendix Fig. 10). Extracellular matrix disruption was highlighted by increased intensity of B-CHP within the superficial layer of the condyle, indicating heightened collagen remodeling (Fig. 3S, U, W; Appendix Fig. 11). Scoring of the phenotype in safranin O–stained sections per the Osteoarthritis Research Society International scale showed grade 6 osteoarthritis in all *FSP1-Cre;DTA* mice at 16 wk (Fig. 3T, V, X). Loss of the most superficial layers of the condyle therefore led to osteoarthritic remodeling of the TMJ.

### Loss of FSP1-Expressing Cells Affected TMJ Shape and Growth of the Dentary

**Figure 4.**
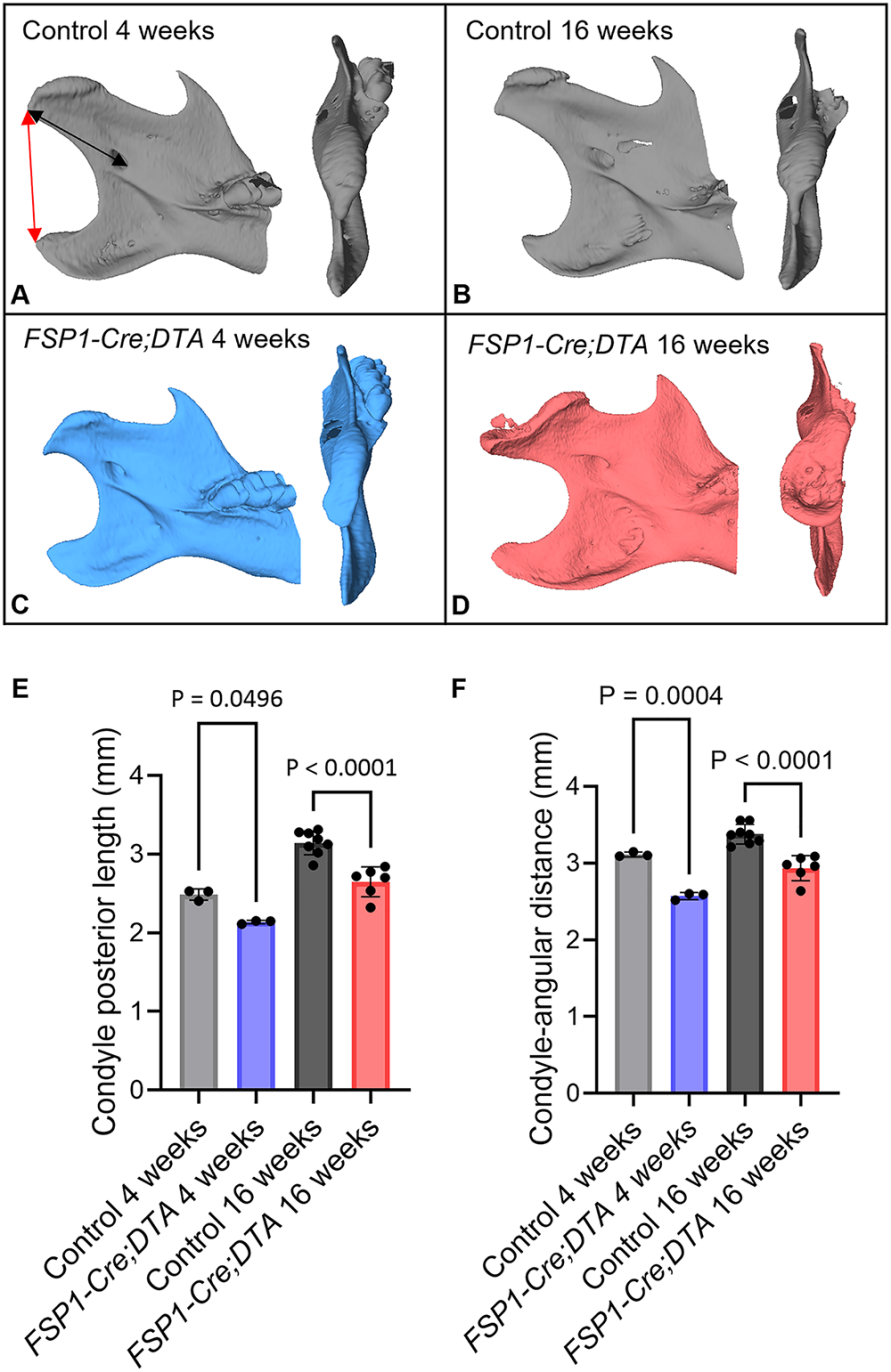
Ablation of *FSP1*-expressing cells affected temporomandibular joint shape and growth of the dentary. (**A**–**D**) Three-dimensional reconstruction micro–computed tomography scans of the condylar proximal mandible in the superior view and internal-lateral view in 4-wk-old (blue, *n* = 3) and 16-wk-old (pink, *n* = 6) *FSP1-Cre;DTA* mice with the Cre-negative *DTA* littermate controls (gray). The black arrow indicates the condyle posterior length, which is the distance from the most caudal point of the condylar process to the most rostral and ventral point of the mandibular foramen (anterior point of lingula). The red arrow indicates the condyle-angular distance, which is the distance from the most caudal point of the condylar process to the tip of the angular process of the mandible. (**E**, **F**) Mean measurements of the condyle posterior length and the condyle-angular distance calculated per Amira software. Error bar indicates SD. One-way analysis of variance followed by Tukey’s multiple-comparison test.

Histologic examination highlighted a significant alteration in the morphology of the condyle after ablation of the FSP1-expressing cells. To assess the impact on the condylar process and dentary bone, we employed micro–computed tomography scanning. Littermate controls aged 4 and 16 wk exhibited a normal TMJ structure, displaying a wide anterior and narrow posterior aspect (Fig. 4A, B). In contrast, in 4-wk-old *FSP1-Cre;DTA* mice, a significant enlargement of the condylar head was observed in the posterior orientation (Fig. 4C). By 16 wk, the posterior condyle was further enlarged, with the condyle having a concave surface associated with ectopic mineralized tissue (Fig. 4D). The growth of the dentary was affected by loss of the FSP1-expressing cells, with significant decreases in length from the condyle to the lingula and between the condyle and angular process (Fig. 4E, F). Loss of the superficial layer thus affected the normal growth of the dentary.

### FSP1 Expression Was Negatively Regulated by Canonical Wnt Activity In Vivo, Resulting in Loss of Cartilage

**Figure 5.**
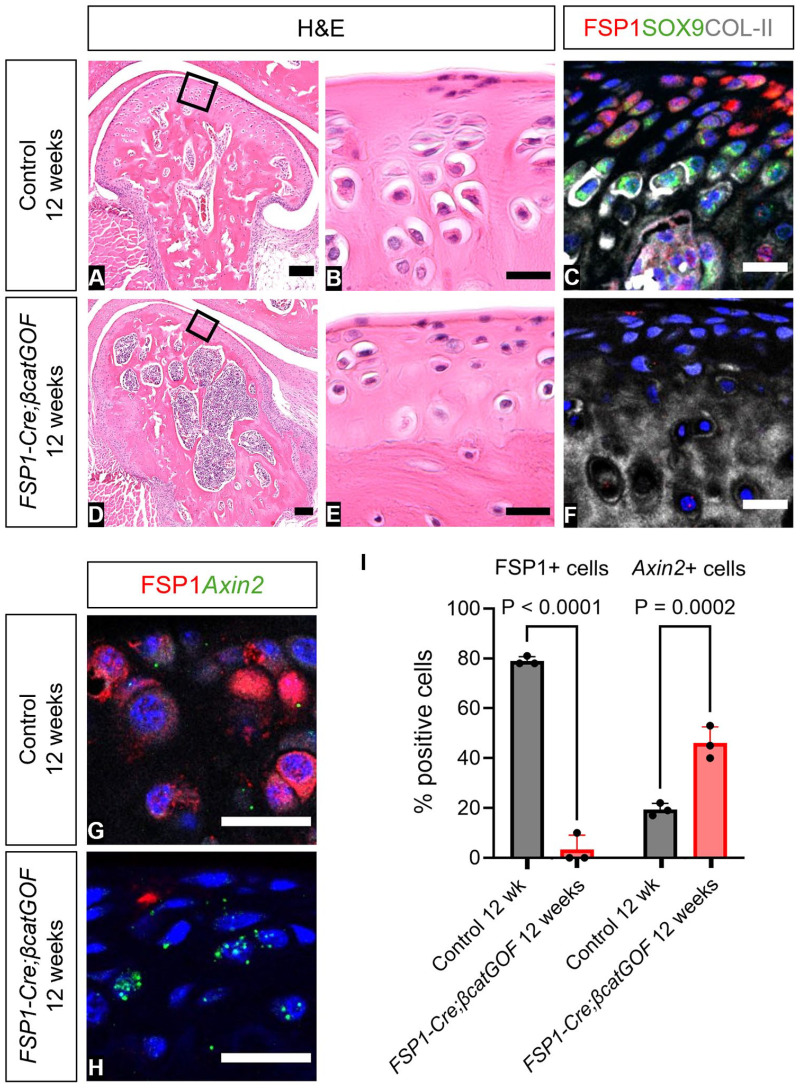
FSP1 expression is negatively regulated by canonical Wnt activity, resulting in loss of temporomandibular joint hyaline cartilage. (**A**–**F**) H&E and immunofluorescence staining in 12-wk-old *FSP1-Cre;βcatGOF* (*n* = 3) and Cre-negative *βcatGOF* littermate controls. (A, D) H&E staining shows condyle morphology. (B, E) The box shows a zoomed-in image of the superficial layer of the condyle. (C, F) Immunofluorescence staining for FSP1 (red), SOX9 (green), COL-II (gray), and DAPI (blue). (**G**, **H**) Representative images of immunofluorescence with RNAscope staining for FSP1 (red), *Axin2* (green), and DAPI (blue) in 12-wk-old *FSP1-Cre;βcatGOF* and Cre-negative *βcatGOF* littermate controls. (**I**) FSP1-expressing cells and *Axin2*-positive cells were quantified by Qupath software. Error bar indicates SD. *n* = 3. Two-way analysis of variance followed by Sidak multiple-comparison test. Scale bars: B, C, E, F, G, and H, 20 µm; A and D, 100 µm. *βcat*, β-catenin; FSP1, fibroblast-specific protein 1; GOF, gain of function; H&E, hematoxylin and eosin.

Loss of FSP1 expression after stimulation of the Wnt pathway in culture suggested that Wnt activity inhibited FSP1 (Fig. 2G–I). To test this in vivo, we utilized *FSP1-Cre*–driven β-catenin gain-of-function mice to constituently activate Wnt/β-catenin signaling in FSP1-expressing cells. *FSP1-Cre;βcatGOF* mice aged 12 wk displayed a major reduction of condylar cartilage, as evidenced by severe osteoarthritic changes when compared with Cre-negative *βcatGOF* littermates (Fig. 5A, B, D, E). The condylar surface was disrupted, with loss of FSP1- and SOX9-expressing cells in the superficial and prechondroblastic zones (Fig. 5C, F). To confirm β-catenin function in *FSP1-Cre;βcatGOF* mice, RNAscope was used to quantify FSP1-expressing and *Axin2*-positive cells, revealing an increase in *Axin2* expression associated with downregulation of FSP1 expression (Fig. 5G–I, Appendix Fig. 12). Loss of canonical Wnt signaling in the condyle postnatally is therefore essential for upregulation of FSP1 and maintenance of the layers of the condyle.

## Discussion

We propose FSP1 as a marker for the superficial fibroblast layers of the mature condyle. FSP1 was induced postnatally, starting in the most superficial layer and later extending to the SOX9-expressing chondrogenic zone of the condyle. Numbers of FSP1-expressing cells increased during development with a sharp rise and expansion of the domain around tooth eruption (Lungová et al. 2011). This suggests that mechanical force may act as a trigger for upregulation. The FSP1-expressing cells could be considered a stem/progenitor population for the postnatal mouse TMJ, with lineage tracing showing that these cels gave rise to all layers of the condyle during postnatal development. Additionally, a subset of FSP1-expressing cells overlapped with other proposed fibroblast stem cell markers, Gli1 and scleraxis (Ochiai et al. 2010; Ma et al. 2021), and were derived from Wnt-responsive *Axin2* cells. A small subset of FSP1-expressing cells was also label retaining, indicating that the FSP1-expressing cells marked stem and progenitor populations. As cells exited the superficial zone and started to differentiate and turn on cartilage markers, expression of FSP1 was downregulated.

Loss of the superficial zone by ablation of FSP1-expressing cells led to severe osteoarthritis of the TMJ. Ablation of FSP1-expressing cells in our model would affect the stem/progenitor cells of the condyle and the superficial synovial cells. Our phenotype was similar to that reported in the *Pgr4* mutant mice, where lubricin production is prevented, resulting in defects in the synovial fluid (Bechtold et al. 2016). In these mutants, the presence of SOX9-expressing cells, coupled with abnormalities in cellular integrity and morphology, led to the formation of osteophytes over time (Bechtold et al. 2016). Loss of the FSP1-expressing cells led to changes in SOX9, extensive remodeling of the extracellular matrix, and ectopic cartilage formation. Chondrocytes play a pivotal role in maintaining cartilage matrix homeostasis, and any compromise in their activity and survival can disrupt this delicate balance, hastening the progression of osteoarthritis (Embree et al. 2010; Lu et al. 2022). Interestingly, recent research has shown that FSP1-expressing cells are the major contributors to collagen I in the bone with *FSP1-Cre* mice (Chen et al. 2021). When collagen I was deleted in FSP1-expressing cells, it led to osteogenic imperfecta–like symptoms in adult mice, characterized by spontaneous fractures and impaired bone healing (Chen et al. 2021).

Expression of FSP1 increased postnatally at the same time as active Wnt signaling was downregulated in the superficial zone. Sustained Wnt signaling in this layer led to loss of FSP1 in vitro and in vivo, highlighting the importance of reducing Wnt signaling in this layer for maintenance of the stem/progenitor population. Previous studies using conditional activation of β-catenin mice have demonstrated TMJOA-like phenotypes, such as cartilage degradation, upregulation of collagen X, decreased cell proliferation, and increased apoptosis (Hui et al. 2018). Excessive Wnt signaling has also been shown to disrupt fibrocartilage homeostasis, causing degenerative changes that lead to the deterioration of the fibrocartilage stem cell population (Embree et al. 2016). Similarly, we have shown that constitutive Wnt/β-catenin signaling in the FSP1 population led to an osteoarthritic phenotype, providing a mechanism for the osteoarthritis phenotype previously observed. In summary, our research highlights the importance of the FSP1-expressing superficial zone and the interplay between FSP1 and Wnt signaling and provides valuable models for investigating TMJOA.

## Author Contributions

T. Tuwatnawanit, contributed to design, data acquisition, analysis, and interpretation, drafted the manuscript; W. Wessman, D. Belisova, contributed to data acquisition, and analysis, critically revised the manuscript; Z. Sumbalova Koledova, contributed to data acquisition, critically revised the manuscript; A.S. Tucker, contributed to conception, design, data interpretation, critically revised the manuscript; N. Anthwal, contributed to conception, design, data acquisition, analysis, and interpretation, critically revised the manuscript. All authors gave final approval and agree to be accountable for all aspects of the work.

## Supplemental Material

sj-docx-1-jdr-10.1177_00220345251313795 – Supplemental material for FSP1/S100A4-Expressing Stem/Progenitor Cells Are Essential for Temporomandibular Joint Growth and Homeostasis Supplemental material, sj-docx-1-jdr-10.1177_00220345251313795 for FSP1/S100A4-Expressing Stem/Progenitor Cells Are Essential for Temporomandibular Joint Growth and Homeostasis by T. Tuwatnawanit, W. Wessman, D. Belisova, Z. Sumbalova Koledova, A.S. Tucker and N. Anthwal in Journal of Dental Research
