# Supplementary material for: FSP1/S100A4-Expressing Stem/Progenitor Cells Are Essential for Temporomandibular Joint Growth and Homeostasis
Source: J Dent Res. 2025 Feb 14;104(5):551–60. doi: 10.1177/00220345251313795 (PMC12000630; doi:10.1177/00220345251313795)
Supplement: sj-docx-1-jdr-10.1177_00220345251313795 – Supplemental material for FSP1/S100A4-Expressing Stem/Progenitor Cells Are Essential for Temporomandibular Joint Growth and Homeostasis [file sj-docx-1-jdr-10.1177_00220345251313795.docx]

| **Mouse** | **MGI Alleles** | **Explanation** | **Housed** |
| --- | --- | --- | --- |
| ***FSP1-Cre;R26RmTmG* (*mTmG*) mice** | ***Tg(S100a4-cre)1Egn***  **MGI: 3712292** | **FSP1 expression drives Cre, which labels cells and their progeny with cell membrane GFP.** | **Laboratory Animal Breeding and Experimental Facility of the Faculty of Medicine, Masaryk University, Brno, Czech Republic** |
|  | ***Gt(ROSA)26^Sortm4(ACTB-tdTomato,-EGFP)Luo^***  **MGI: 3716464** |  |  |
| ***FSP1-Cre;R26RDTA* (*DTA*) mice** | ***Tg(S100a4-cre)1Egn***  **MGI: 3712292** | **FSP1 expression drives Cre, which result in expression of diphtheria toxin and kill the cells.** |  |
|  | ***Gt(ROSA)26Sor^tm1(DTA)Jpmb^***  **MGI: 3610389** |  |  |
| ***FSP1-Cre;Ctnnb1^ex3(loxP)^***  **(*βcatGOF*) mice** | ***Tg(S100a4-cre)1Egn***  **MGI: 3712292** | **FSP1 expression drives Cre, which result in expression of stabilized active beta-catenin protein.** |  |
|  | ***Ctnnb1^tm1Mmt^***  **MGI:1858008** |  |  |
| ***Axin2-CreERT2;tdTom* mice** | ***Axin2^tm1(cre/ERT2)Rnu^***  **MGI:5433373** | **Axin2 expression drives tamoxifen-activated Cre, which label cells and their progeny with RFP.** | **King’s College London, London, UK** |
|  | ***Gt(ROSA)26Sortm9(CAG-tdTomato)Hze***  **MGI:3809523** |  |  |
| **CD1 mice** | **n/a** | **Strain of wildtype mice** |  |
| ***Mesp1Cre;tdTom*mice** | ***Mesp1^tm2(cre)Ysa^***  **MGI:2176467** | **Mesoderm marker (Mesp1) expression cell drives tamoxifen activated Cre, which label cells and their progeny with RFP.** |  |
|  | ***Gt(ROSA)26Sortm9(CAG-tdTomato)Hze***  **MGI:3809523** |  |  |

**Appendix Table1: Mouse models.**

| **Reagent** | **Source** | | **Identifier** |
| --- | --- | --- | --- |
| **Antibodies, probes, and reagents** | | | |
| **Rabbit-Anti-Fsp1/S1000A4 (1:350)** | | **EMD Millipore Corp, USA** | **07-2274; Lot 3953647, Lot 4095712** |
| **Goat-Anti-hSox9 (1:200)** | | **R&D systems** | **AF3075; Lot WIL0523091** |
| **Mouse-Anti-Col2 (1:50)** | | **DSHB** | **CIIC1-s** |
| **Chick-Anti-GFP antibody (1:300)** | | **abcam** | **Ab13970; 1018753-6** |
| **Goat-Anti-RFP antibody (1:300)** | | **Antibodies.com** | **A121675; Lot 40111** |
| **Rat-Anti-BrDU (1:500)** | | **abcam** | **AB6326; 1009715-6** |
| **Collagen Hybridizing Peptide, Biotin Conjugate (B-CHP)** | | **3Helix** | **BIO60** |
| **Alexa Fluor® 647 Streptavidin** | | **BioLegend®** | **Cat: 405237; Lot B374387** |
| **Alexa Fluor™ 647 donkey anti-rabbit** | | **Invitrogen** | **A31573; Lot 2544598** |
| **Alexa Fluor™ 568 donkey anti-goat** | | **Invitrogen** | **A11057; Lot 2563881** |
| **Alexa Fluor™ 488 donkey anti-mouse** | | **Invitrogen** | **A21202; Lot 2563848** |
| **Alexa Fluor™ 488 donkey anti-chicken** | | **Invitrogen** | **A78948; Lot 2622389** |
| **Alexa Fluor™ 488 donkey anti-rat** | | **Invitrogen** | **A21208; Lot 2668657** |
| **Mm-Axin2 probe** | | **RNAscope®** | **400331-C3; Lot 23317A, Lot 24270C** |
| **Mm-Lgr5 probe** | | **RNAscope®** | **312171-C2; Lot 24026B** |
| **Mm-Gli1 probe** | | **RNAscope®** | **311001-C2; Lot 23131B** |
| **Mm-Scx probe** | | **RNAscope®** | **439981-C1; Lot 24085B** |
| **Mm-S100a4 probe** | | **RNAscope®** | **412971-C1; Lot 24281B** |
| **Probe Diluent** | | **RNAscope®** | **300041; Lot 2016050** |
| **TSA buffer** | | **RNAscope® Multiplex** | **322809; Lot 2014309, Lot 2025987** |
| **RNAscope® Multiplex Fluorescent Detection Reagents Kit v2** | | **Advanced Cell Diagnostics** | **323110, Lot 2023446** |
| **H2O2 and Protease reagents** | | **RNAscope®** | **322381; Lot 2013430** |
| **Chondroitinase ABC from Proteus vulgaris** | | **SIGMA-ALDRICH, Co** | **C2905-10UN; Lot 0000225027, Source 0000223911** |

**Appendix Table2: Antibodies, probes, and reagents.**


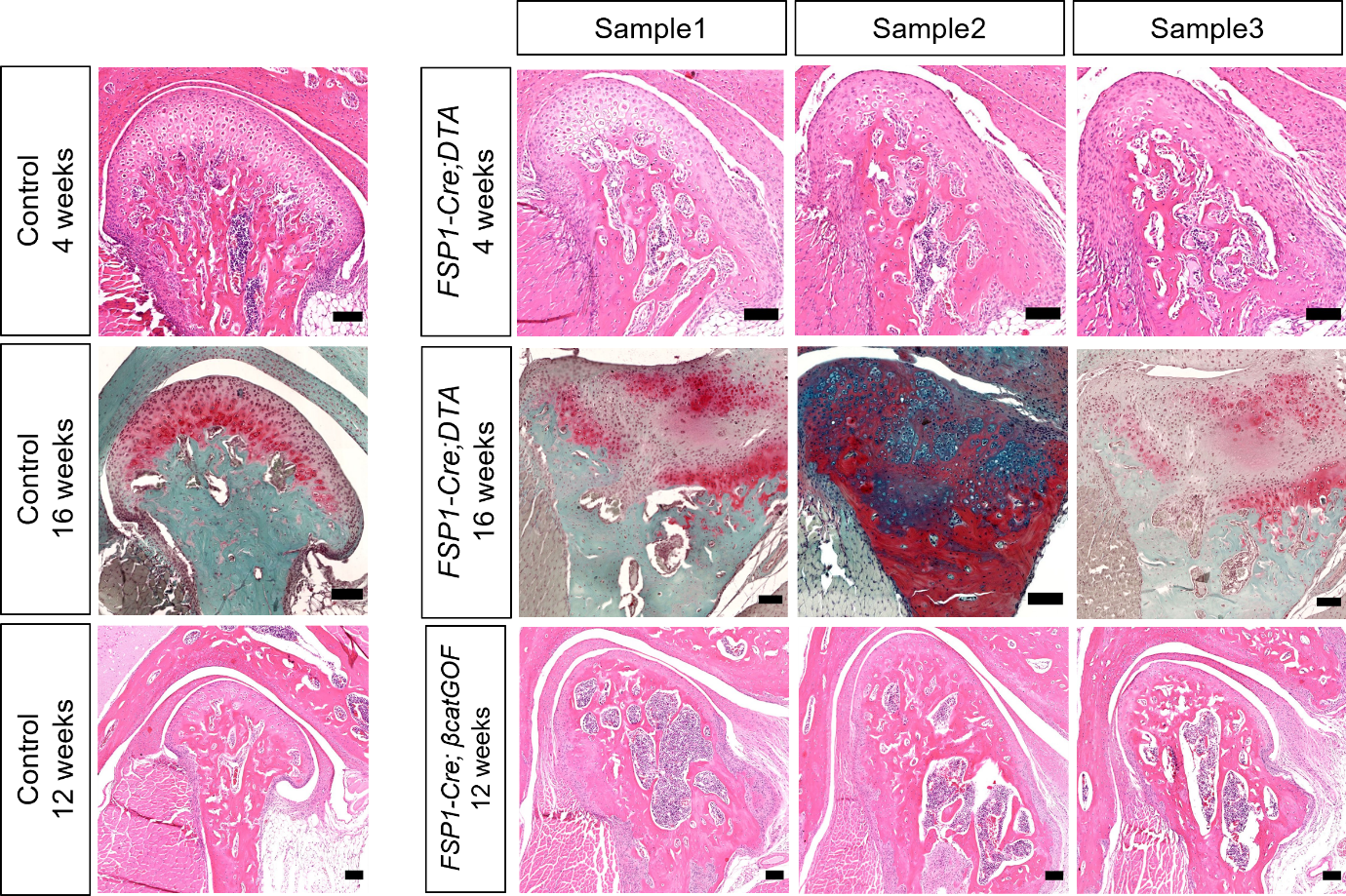


**Appendix Figure 1: Histological changes in mutant TMJs.**

Histology staining of mouse models**:** 4-week-old *FSP1-Cre;DTA* mice, 16-week-old *FSP1-Cre;DTA* mice, and 12-week-old *FSP1-Cre;βcatGOF* mice (n=3). scale bar: 100 µm.


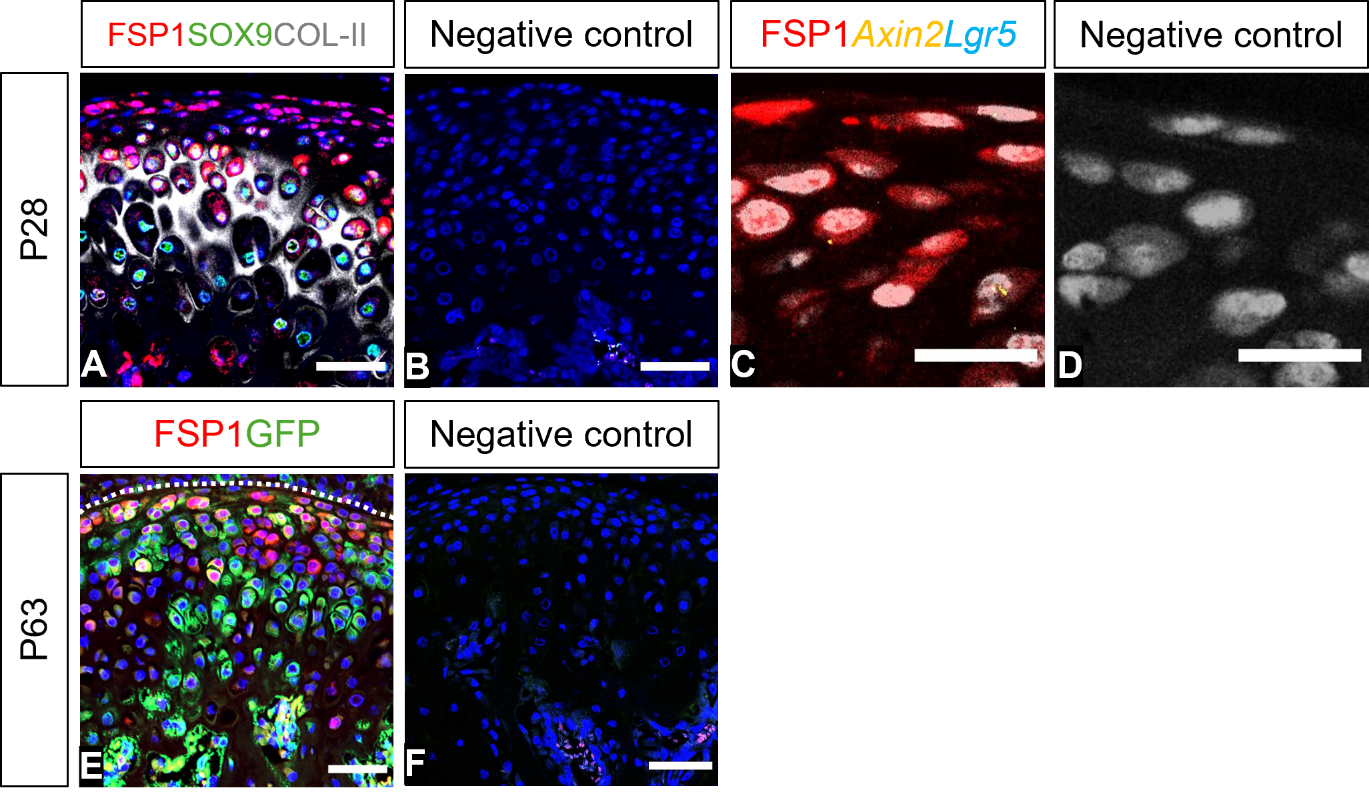


**Appendix Figure2: Immunofluorescence and RNAscope control slides.**

(A) Immunofluorescence staining for FSP1 (red), SOX9 (green), COL-II (grey), and DAPI (blue) in P28 CD1 mice. (B) a negative control slide of immunofluorescence staining in A. (C) Dual immunofluorescence and RNAscope staining for FSP1 protein (red), *Axin2* mRNA (yellow), *Lgr5* mRNA (cyan), and DAPI (grey) in P28 CD1 mice. (D) a negative control slide of dual immunofluorescence and RNAscope staining in C. (E) Immunofluorescence staining for FSP1 (red), GFP (green), and DAPI (blue) in P63 *FSP1-Cre;mTmG* mice. (E) Dash line indicates condyle surface. (F) a negative control slide of immunofluorescence staining in E. Scale bar A-B, E-F: 50 µm, scale bar in C-D: 20 µm.


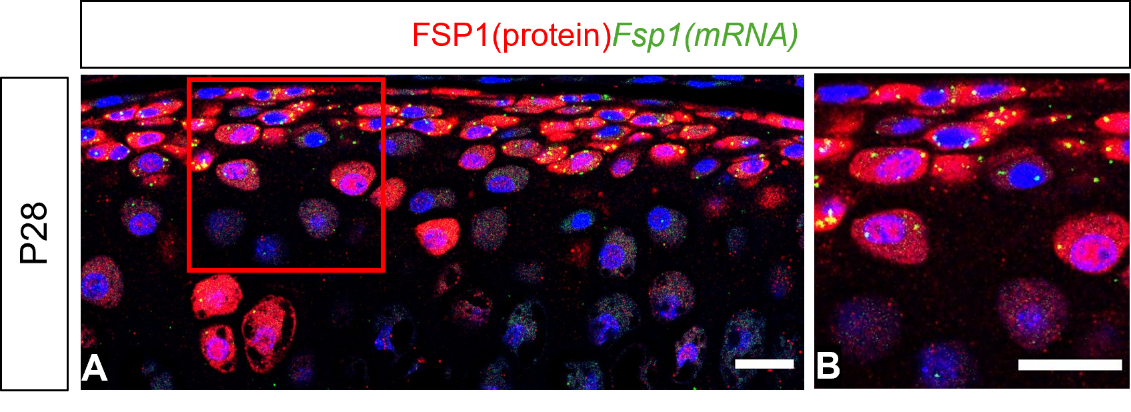


**Appendix Figure3: Co-localisation of FSP1 protein and mRNA.**

1. Dual immunofluorescence and RNAscope staining for FSP1 protein (red), *Fsp1* mRNA (green), and DAPI (blue) in P28 CD1 mice. (B) The box shows a zoomed-in image. Scale bar A-B: 20 µm


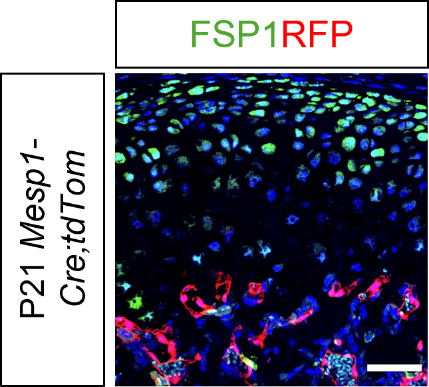


**Appendix Figure4: FSP1 cells are not derived from the mesodermal lineage.** Immunofluorescence staining for FSP1 (green), RFP (Red fluorescent protein) (red), and DAPI (blue) in P21 *Mesp1Cre;tdTom*mice. Scale bar: 50 µm


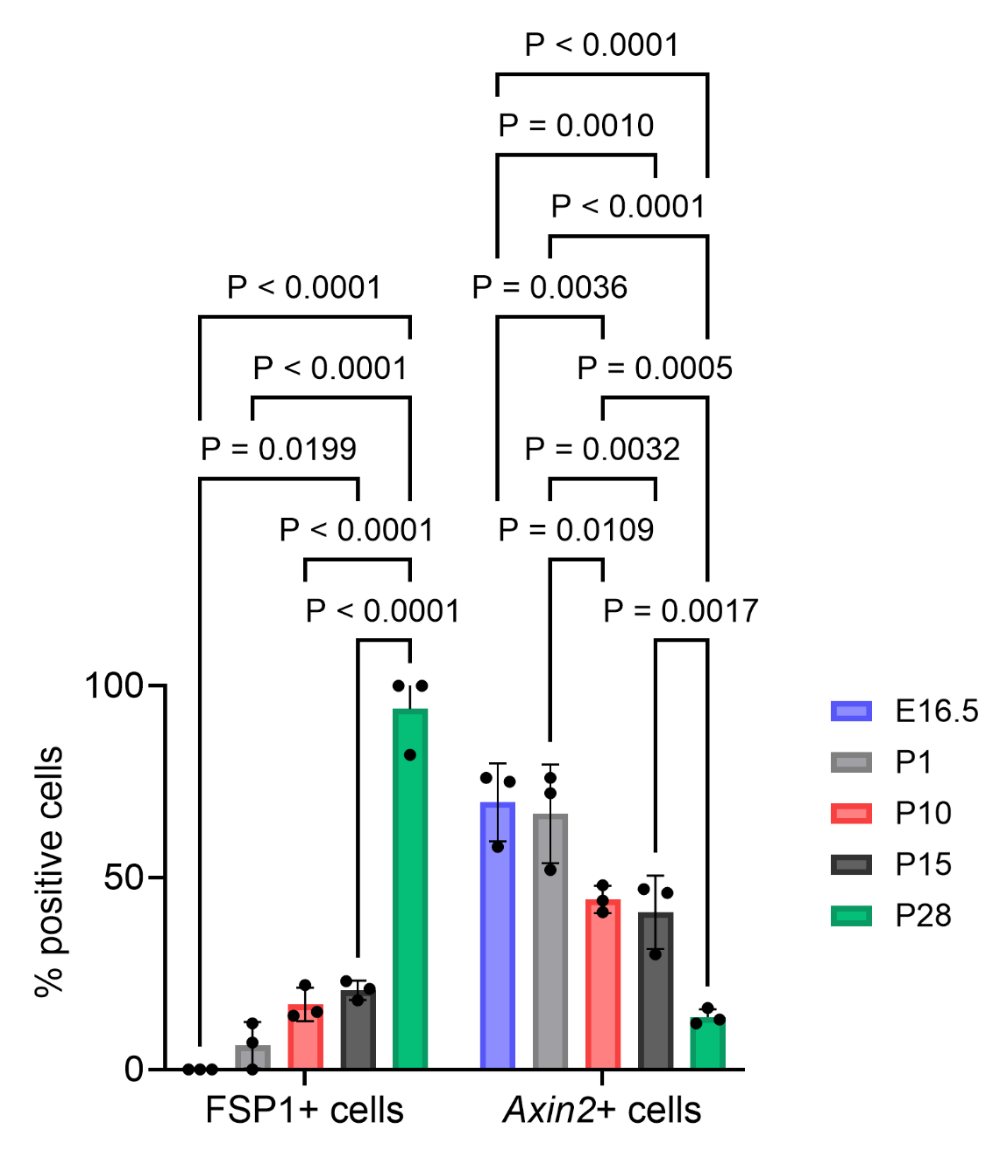


**Appendix Figure5: Quantification of FSP1-expressing cells and *Axin2*-positive cells during condyle growth.**

Error bar = ±S.D.; n=3; two-way ANOVA flowed by Tukey’s multiple comparisons test.


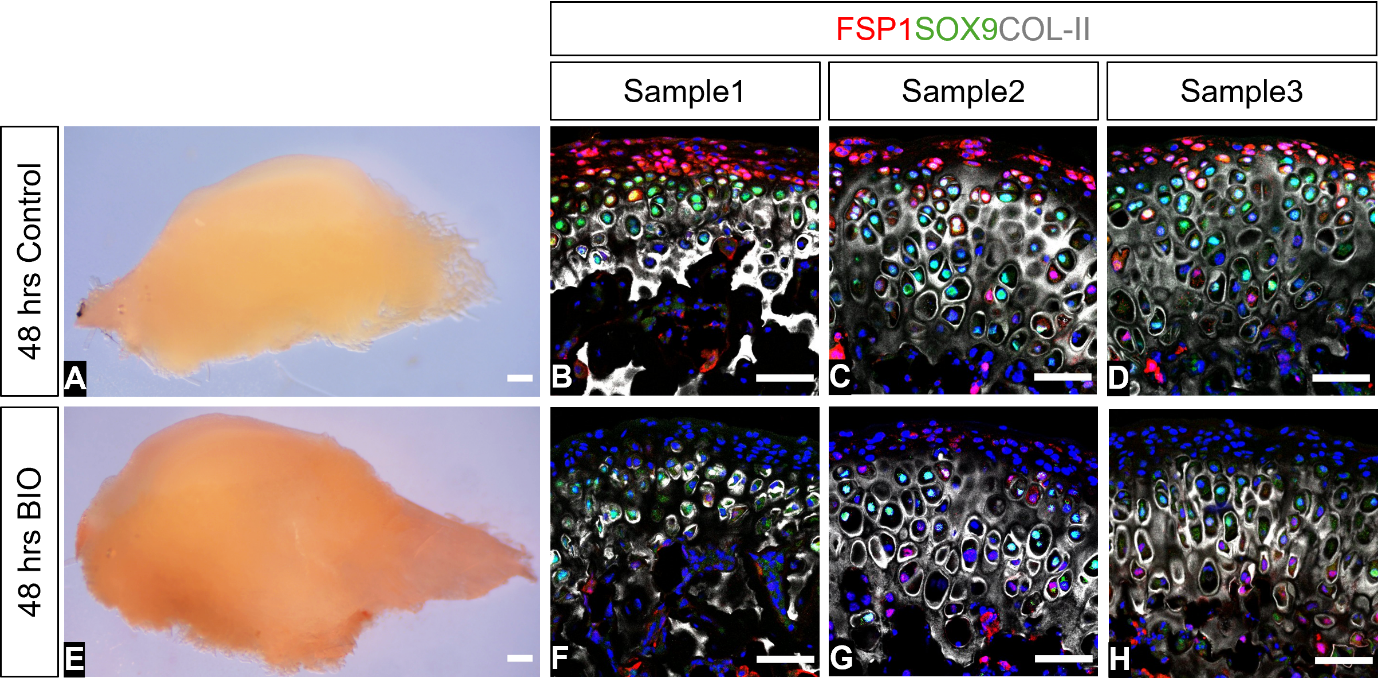


**Appendix Figure6: Impact of upregulation of the canonical Wnt pathway on FSP1 expression.**

(A,E) P21 CD1 condylar heads were dissected and contralateral sides were cultured +/- 50uM BIO in DMSO in Advanced DMEMF12 following a Trowell method. (B-D,F-H) Immunofluorescence staining for FSP1 (red), SOX9 (green), COL-II (grey), and DAPI (blue). Scale bar in A,E: 200 µm, scale bar in B-D and F-H: 50 µm


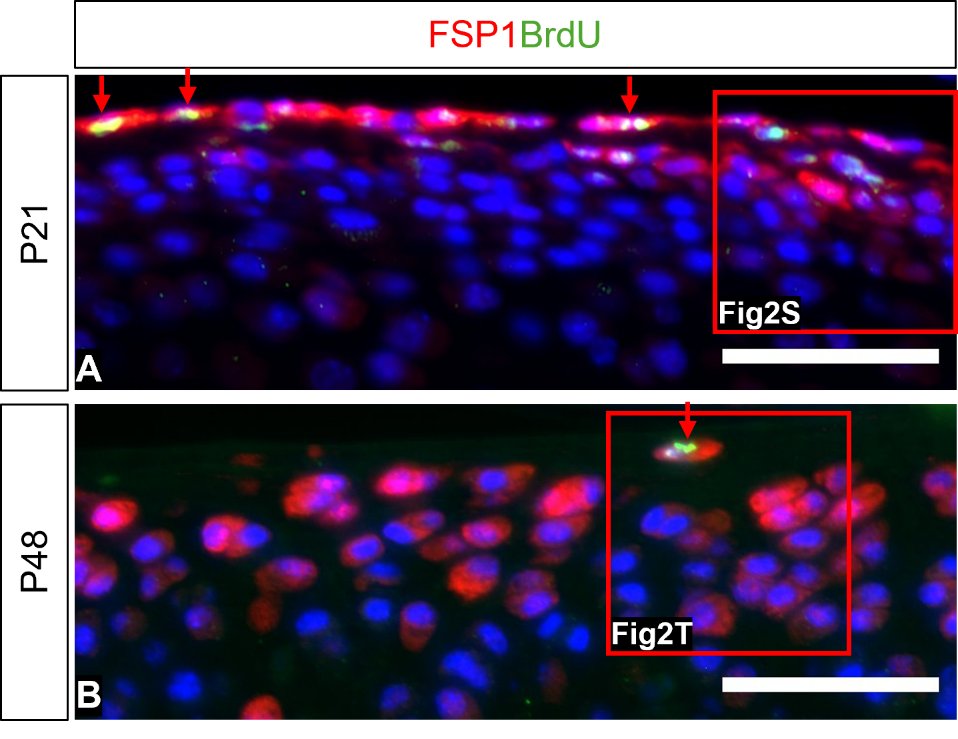


**Appendix Figure7: BrdU label retaining cells co-express FSP1.**

BrdU was administered to CD1 mice at E17.5, E18.5. Immunofluorescence staining for BrdU (green), FSP1 (red), and DAPI (blue) in P28 (A) and P48 (B) CD1 mice. Arrows indicate FSP1-expressing cells (red) co-stained with BrdU LRCs (green). Scale bar in A,B: 50 µm


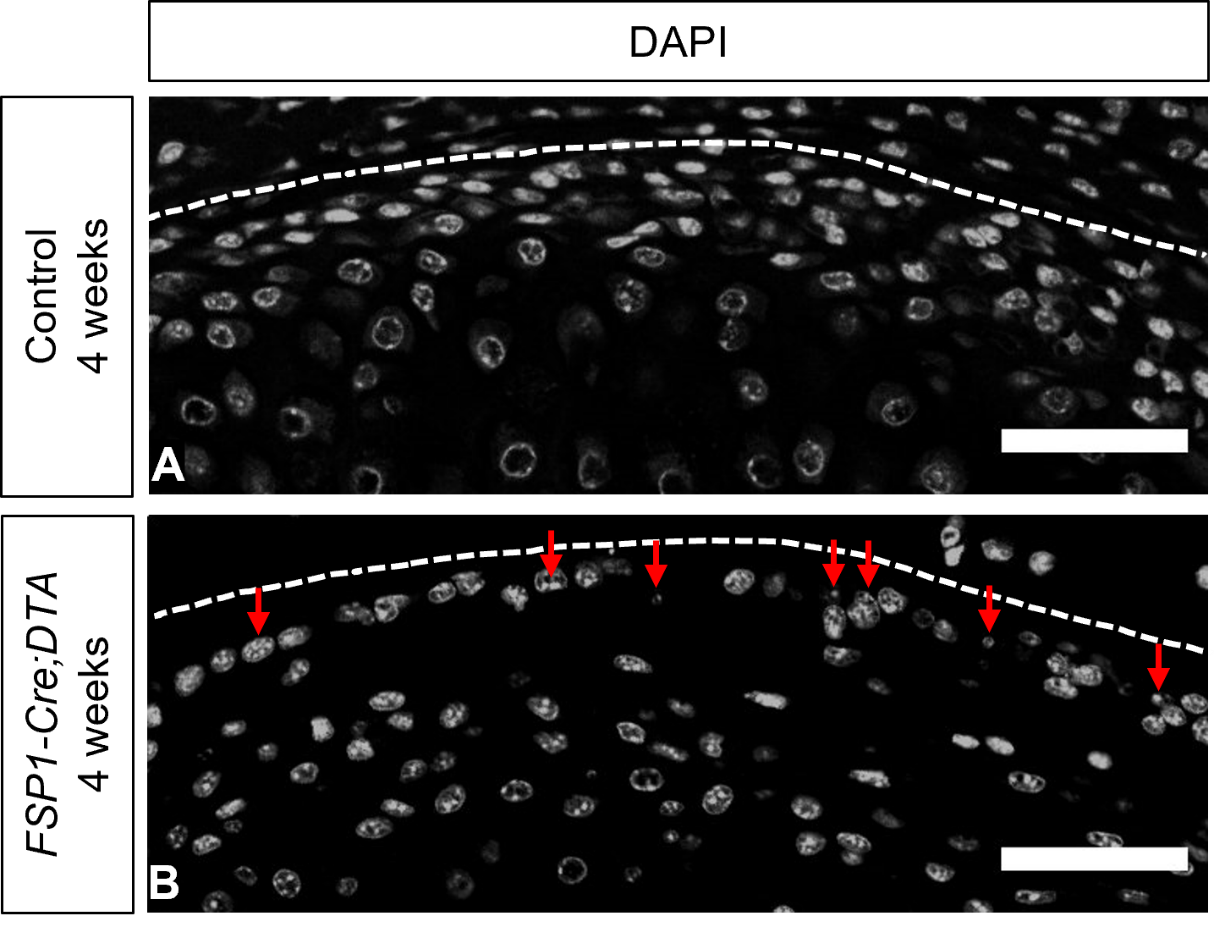


**Appendix Figure 8: Apoptotic bodies in the superficial layers of *FSP1-Cre;DTA* mice.**

(A) DAPI (grey) staining in 4-week-old *FSP1-Cre;DTA* mice, (B) along with the Cre-negative *DTA* littermate controls. Dash line indicates condyle surface. Arrows indicate apoptotic bodies. Arrow heads Scale bar in A,B: 50 µm


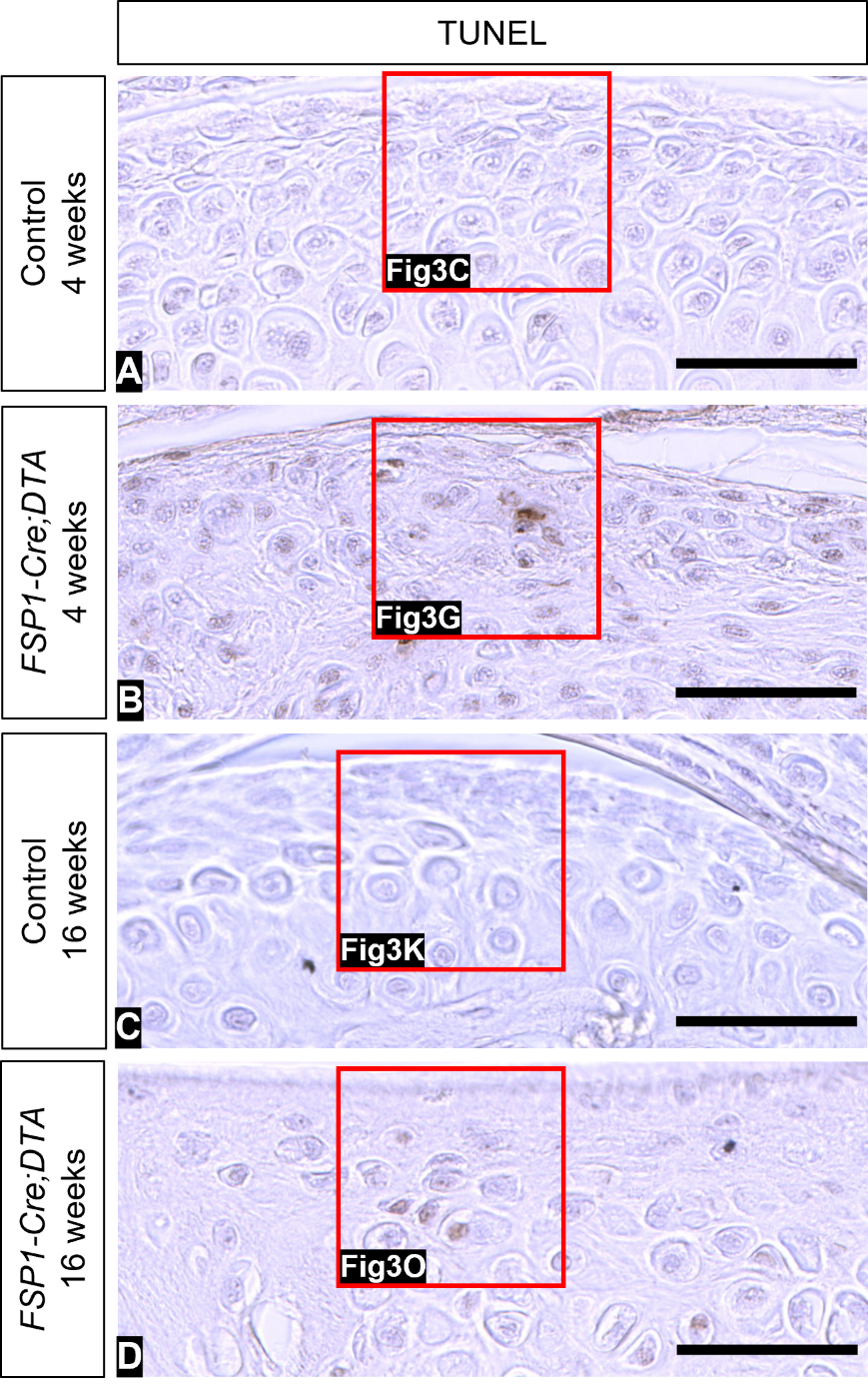


**Appendix Figure9: TUNEL assay in *FSP1-Cre;DTA* mice.**

(A-D) TUNEL assay in 4-week-old and 16-week-old *FSP1-Cre;DTA* mice, respectively, along with the Cre-negative *DTA* littermate controls. Scale bar in A-D: 50 µm


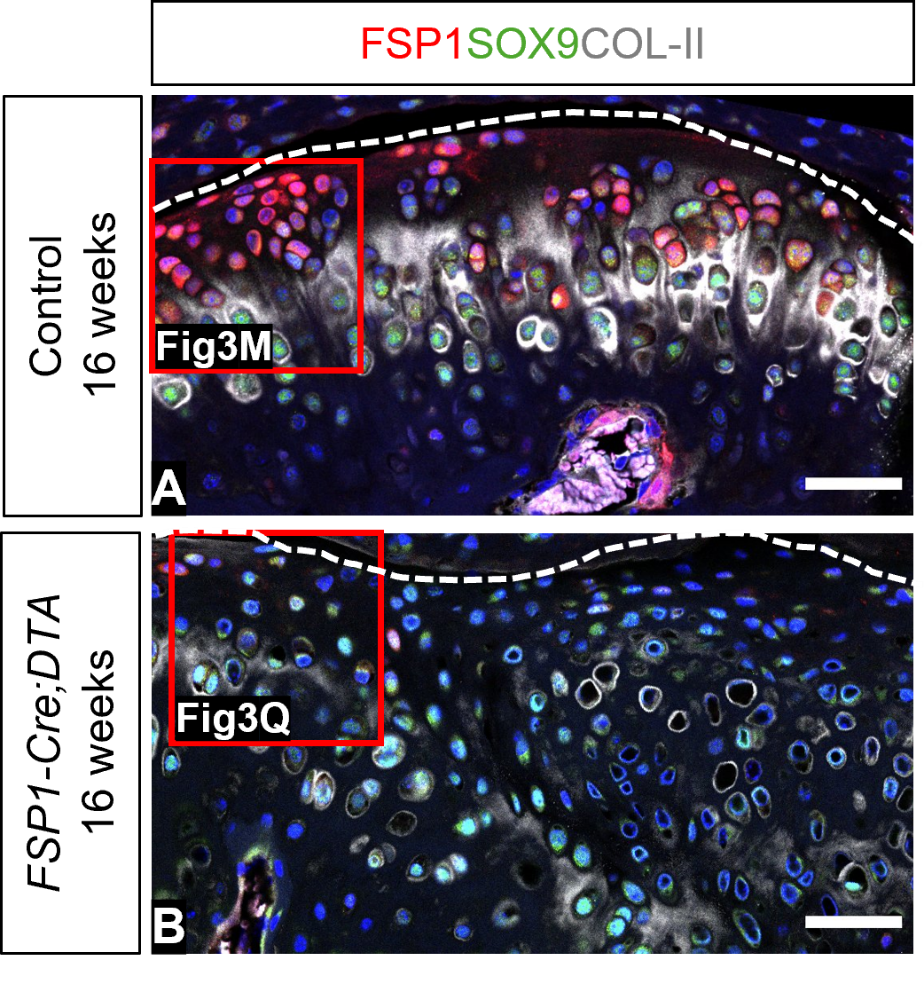


**Appendix Figure10: Removal of the superficial layers by *FSP1-Cre-*driven DTA led to loss of condylar structure**

(A-B) Immunofluorescence staining for FSP1 (red), SOX9 (green), COL-II (grey), and DAPI (blue) in 16-week-old *FSP1-Cre;DTA* mice along with the Cre-negative *DTA* littermate controls. Scale bar in A,B: 50 µm


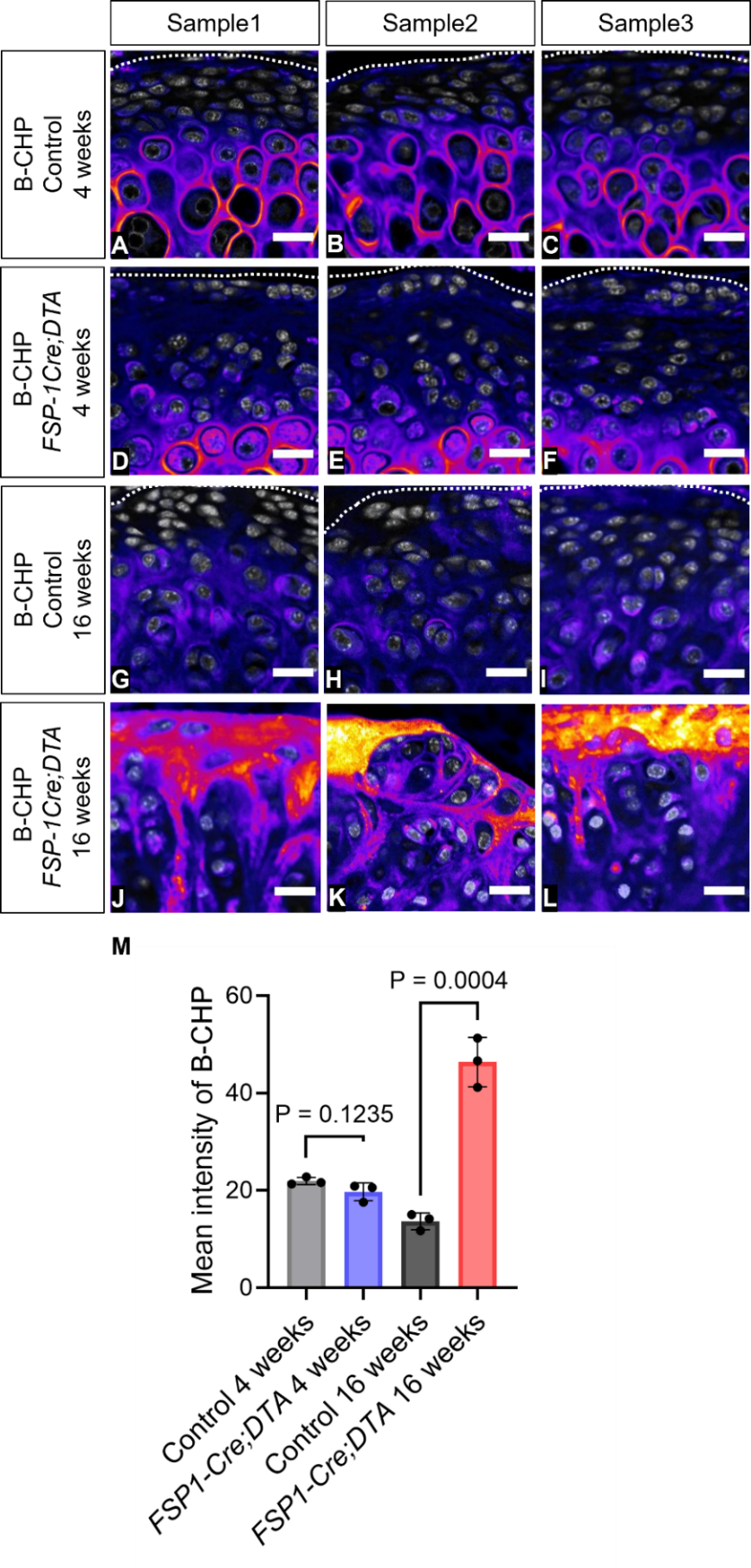


**Appendix Figure11: Collagen remodelling in *FSP1-Cre;DTA* mice**.

(A-L) Immunofluorescence staining for B-CHP (fire LUT) and DAPI (grey) in 4-week-old and 16-week-old *FSP1-Cre;DTA* mice, respectively, along with the Cre-negative *DTA* littermate controls. (M) Mean intensity of B-CHP was analysed using ImageJ software. Error bar = ±S.D.; n=3 unpaired t test. Scale bar in A-L: 20 µm.


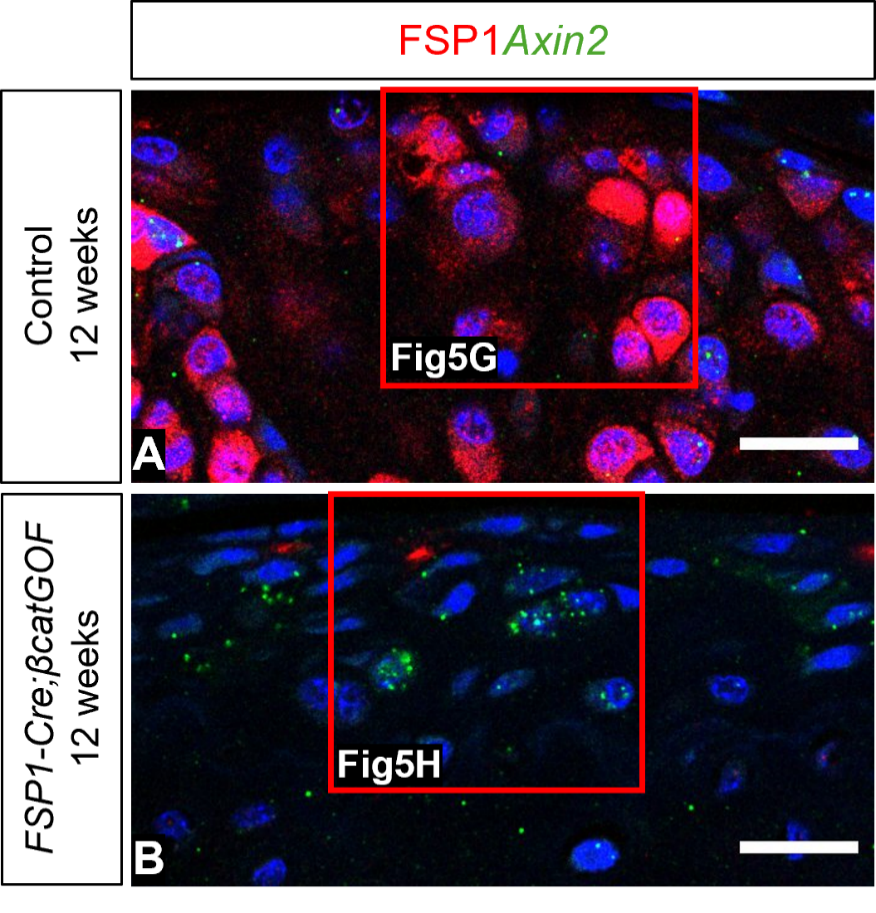


**Appendix Figure12: FSP1 expression is negatively regulated by canonical Wnt activity**

(A-B) Immunofluorescence with RNAscope staining for FSP1 (red), *Axin2* (green), and DAPI (blue) in 12-week-old *FSP1-Cre;βcatGOF* and Cre-negative *βcatGOF* littermate controls. Scale bar in A,B: 20 µm
